# Biomimicry for Energy-Efficient Building Design: A Bibliometric Analysis

**DOI:** 10.3390/biomimetics7010021

**Published:** 2022-01-24

**Authors:** Niloufar Varshabi, Semra Arslan Selçuk, Güneş Mutlu Avinç

**Affiliations:** 1 Department of Architecture, Faculty of Architecture, Gazi University, Ankara 06560, Turkey; niloofar.varshabi@gmail.com (N.V.); semraselcuk@gazi.edu.tr (S.A.S.); 2 Faculty of Engineering and Architecture, Muş Alparslan University, Muş 49100, Turkey

**Keywords:** biomimicry, energy efficiency, architecture, bibliometric analysis

## Abstract

With the development of the biomimicry approach, new and creative ideas have been established to solve problems in architectural design. In the designs based on this process, “nature” is used as a diverse data source for the transfer of these data to various processes, functions, materials, and structures. The primary purpose of this paper is to explore the development of biomimicry as an architectural approach, with a bibliometric review of research related to biomimicry and energy efficiency. Emphasis on the importance of the need for biomimicry in modern designs is another goal of this study. In this study, articles published in the Web of Science database (2010–2021) were analyzed. VOSviewer and SankeyMATIC software were used to represent the analysis results graphically. According to the results of this study, in addition to the inadequacy of biomimicry research, the need for further research became apparent. This review can serve as a reference for future studies to transfer natural phenomena to architecture in order to solve the problem of efficient energy consumption.

## 1. Introduction

Climate change throughout the world has increased concerns about ecological harm. Greenhouse gas emissions from this activity inflict permanent harm. Global advances in energy efficiency have been dropping since 2015 according to IEA2020. A decrease in energy costs arrived with the COVID-19 pandemic, resulting in a reduction in energy intensity improvement in 2020, less than the revised rates for 2018 and 2019. As a consequence of the analytical studies of energy institutions, buildings account for one-third of this consumption. Buildings are also responsible for 40% of direct and indirect emissions of CO_2_ [1].

The importance of construction in worldwide energy consumption has raised awareness of environmental degradation trends. This rising knowledge has sparked efforts to optimize energy efficiency while minimizing environmental impact. The use of efficient designs reduces the energy consumption of a building during its life cycle and helps solve these challenges by reducing the damage to the environment and human health.

Architects and designers have long been concerned with meeting the challenges of nature and architecture. Owing to advances in technology, nature-based design and construction techniques have led to designs based on biological approaches. These techniques, which appear with various terminologies such as bionics, bio-inspiration, biomimetics, and biomimicry, are mainly based on solving problems by inspiring/learning/developing innovative proposals based on the shape, structure, and systems of nature”. It has been also argued that “designers can be inspired by nature in order to tackle building design challenges, and this has led to a connection between architecture and biomimicry” [2].

Biomimicry’s goal, developing technical advancements and achieving sustainability, appears to be the best ideas of nature, and it imitates them in design processes to solve design problems. There is a rising focus on how organisms adapt to changes in the environment, with the claim that these characteristics might bring new approaches to designing technologies to improve performance [3].

New technologies can improve the energy efficiency of any kind of energy-consuming systems. Likewise, biomimicry is considered an “innovative design strategy for increasing energy-efficient designs” [4], and, thus, energy-efficient built environments. Due to the existence of diverse biological mechanisms, natural processes with low energy consumption can inspire creative solutions for optimal energy consumption. It is also a matter of debate that “bio-inspired design may present the potential for causing a paradigm change in traditional design thinking” [5]. Bio-inspired/bio-informed designs can be solution or problem oriented. In this way, these biological mechanisms are explored for novel approaches. “A problem-based approach to provide a better response to building energy reduction, means that once architects are aware of thermal challenges and want to improve thermal performance using bio-inspired design solutions, their priority is to discover appropriate biological examples” [6].

If scientific analysis studies are conducted conventionally, increasing the number of academic publications may result in various limitations in the research. For this purpose, structuring the analytical research with a new method is helpful when it comes to broadening the area of the investigation. Literature reviews stand out as one of these techniques and play a vital role in bringing together previous studies’ results. With these types of studies, the present observation’s foundation may be successfully utilized; as a result, the research phase is increased and broadened in light of the evaluations to be made regarding research in the relevant field. One of the review procedures that is widely utilized is “bibliometric analysis”. Bibliometric analyses are focused on studying specific aspects of an article’s content or publishing and producing different scientific communication conclusions [7].

As research progresses, terms and ideas become broader, more multidisciplinary, and more complex, and, despite this progress, questions remain unanswered. Within this framework, in this study, bibliometric research was conducted, and some aspects are discussed. Firstly, articles from Web of Science were studied in order to identify bibliometric indicators in this research, such as annual publication analysis, research area analysis, Geographical area evaluation, analysis of authorship, analysis of organizations, citation by sources, co-occurrence of keywords, and content analysis of the selected articles. Then, as given in Section 2, data visualization was performed using VOSviewer and SankeyMatic software. In the Section 3, the bibliometric findings are discussed. As an inference, biologically inspired approaches to building energy efficiency have been introduced by highlighting why and how bio-informed knowledge has been transferred.

## 2. Research and Methodology

The purpose of this study was to survey publications and research directions in biomimicry and architecture utilizing a scientific literature database and science survey analysis. Bibliometric analysis is an effective method for determining the growth and future possibilities of a study topic. Bibliometric methodologies have been used to investigate the growth of research topics, find relationships between logical progress and modifications to the approach, and identify expanding interdisciplinary alliances. The research methodology provides detailed quantitative and statistical scientific information regarding the processes of making decisions.

Today, detailed analyses can be conducted via numerous online databases such as Web of Science, Scopus, and Google Scholar. Additionally, different software tools such as VOSviewer, Citespace, Bibexcel, Gephi, Pajek, Publish or Perish, Ucinet, and Science of Science (Sci2) provide informative data and visualization [8]. The bibliometric data of the search results made with the relevant keywords in the WoS (Web of Science) database were analyzed and visualized with the VOSviewer and Sankey diagram mapping methods, and the relationships, trends, and collaborations between the subjects were revealed through network maps. In addition to primary databases such as WoS, Scopus, EBSCO, and ProQuest used in bibliometric searches, networks such as Google Scholar, ResearchGate, and Academia are also available. The search results in Google Scholar, despite the variety, are not comprehensive, and the results have a different quality load, so the classification of articles for bibliometric work is the responsibility of the researcher. In Google Scholar, updating information related to articles is not accurate, so a bibliometric review of the articles may not have acceptable results. Classification studies were carried out in this study using the Web of Science, which includes indexes such as Sci, Sci, and A&HCI, where the most recent and qualified publications are published.

**Figure 1:**
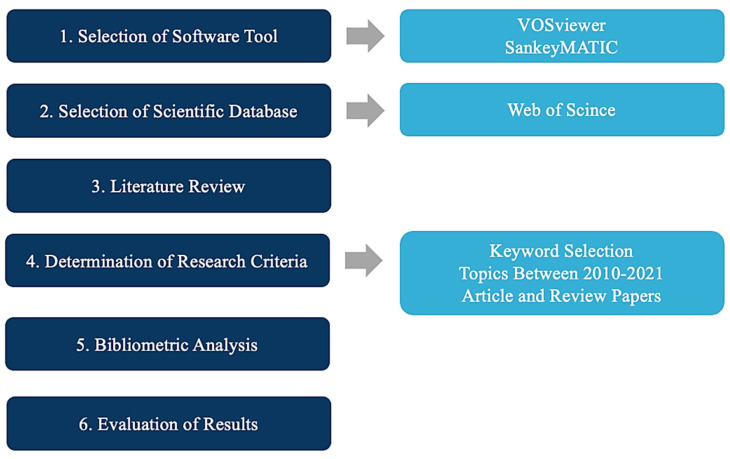
Methodology of the study.

The bibliometric analysis in this research is based on two mechanisms of functional analysis and scientific mapping. Scientific mapping is a visual representation of the conceptual, intellectual, and social structures of research fields [7]. In this method, bibliographic data analysis is possible based on the author, document, source, institution, country, and keywords. In this research, the analysis of co-occurring words was used to map the studies, which is a method used to measure the strength of relationships between publications’ keywords [9]. By analyzing co-occurring words, keywords related to the subject of the articles were obtained. Performance analysis is a method that measures the number of articles and citations published by authors and institutions in this field. In this study, mapping and performance analysis were used together in an integrative way. Bibliographic data from the Web of Science database were analyzed in VOSviewer and SankeyMATIC software. Figure 1 summarizes the study’s methodology.

Web of Science is “a website that offers subscription-based access to several databases that contain detailed citation data for a wide range of academic subjects” [10]. With the help of such an advanced tool, it is possible to track ideas and disciplines over time, with over 1.9 billion citations from 171 million records in the database. Users can also obtain all of the data for entries in a certain field of research based on a provided query phrase [10]. Visualization of similarity (VOS) mapping is a topic-based approach that allows for a comprehensive and clear representation of advancing study disciplines. It can also draw clusters and cooperation networks using vortices connected by lines [8]. The Sankey diagram (SankeyMATIC) demonstrates a wide data relation to visualize multifaceted connections. It is a type of diagram used to visualize the quantities and flows of assets in proportion to each other. The width of the lines or nodes in the diagram is used to express the size of the data. In this context, the size of the line or node also expresses the size of the flow amount [11].

Biomimicry is an approach that is widely used in material science, architecture, and engineering, etc. In this work, we integrated the network, group, and bibliometric studies of the scientific literature in the context of biomimicry and building energy efficiency by addressing some questions:

- How many peer-reviewed articles in the field of biomimicry and energy efficiency in architecture are available, and what is the overall growth?
- What are the main scientific and technological topics addressed by this data analysis?
- Which articles have received the most citations?
- What are the most active journals for this type of research?
- Which research institutes are at the forefront of this field of study?
- Which countries are engaged in promoting growth in this field?
- Who are the main authors, and who is working with whom?

### Data-Gathering Method

The study used articles screened for “titles, abstracts, and keywords” in English from the WoS (Web of Science) database. “Biomimetic(s)”, “biomimicry”, or “bio-inspired” and “thermoregulation”, “energy efficiency”, or “envelope/façade” were used as keywords to analyze trends in biomimicry between 2010 and 2021.

A total of 732 items in the specified period were identified. The records were divided into eight classes: articles (569; 77%), review articles (94; 12%), proceedings papers (70; 9%), book chapters (15; 2%), and others such as early access papers, editorial materials, and meeting abstracts. A total of 251 of the items were ‘open access’ (34%); the remainder were available through subscription.

Articles (148) and review papers (30) under the categories of architecture, construction building technology, energy fuels, ecology, environmental science, interdisciplinary engineering, and multidisciplinary papers were selected for a detailed analysis. A total of 53 research and review articles about biomimicry and energy efficiency in architecture were obtained.

## 3. Results and Discussion

The data collected from the WoS database were analyzed based on the year of publication, research area, geographical area, authorships, citations, and the transition of biological phenomena to the architectural products studied.

### 3.1. Annual Publication Analysis

**Figure 2:**
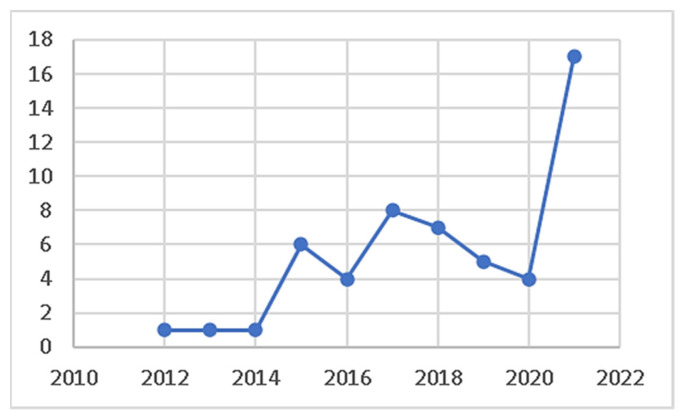
Annual number of publications related to biomimicry and energy efficiency.

Figure 2 presents the distribution of publications by year, demonstrating that research in the relevant subject has intensified, and the number of articles has increased in the last five years. In 2021, 17 articles with the specified qualities were published, and the highest number was reached. Based on the results, more comprehensive research on biomimicry and energy efficiency is needed. On the other hand, the increasing number of articles indicates a growing interest in this field.

### 3.2. Research Area Analysis

**Figure 3:**
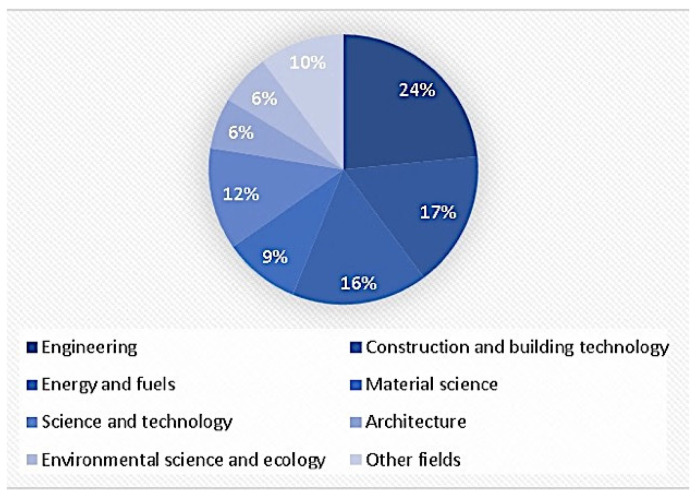
Subject area distribution of the related literature in biomimicry and energy efficiency.

The pie chart in Figure 3 indicates the thematic classification of biomimicry and energy efficiency in buildings. According to the chart, “engineering” is the most researched topic (24%). Substantial studies were conducted in” building technology, energy studies, materials science, and environmental studies”. Additionally, 10% of the total research area distribution consists of studies in computer science, robotics, thermodynamics, urban studies, physics, and chemistry.

The highest number of publications is in engineering (23), and construction and building technology (16) is in second place. Additionally, the input from the other areas has been shown clearly (Table 1).

### 3.3. Geographical Area Evaluation

**Figure 4:**
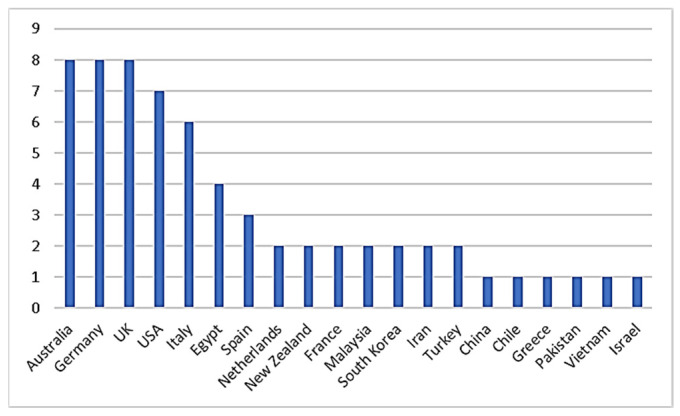
Countries and the number of publications.

Figure 4 shows the countries with publications in the relevant field. While 20 countries contributed to the 52 articles examined, only 5 countries published 6 or more, which shows the inadequacy of such research in many countries. In terms of the number of publications on biomimicry and energy efficiency, Australia, Germany, and the United Kingdom are at the top of the list, then Italy and United States follow them. These five countries account for more than 70% of the publications in this area.

**Figure 5:**
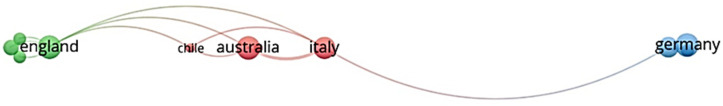
Collaboration network between countries.

**Figure 6:**
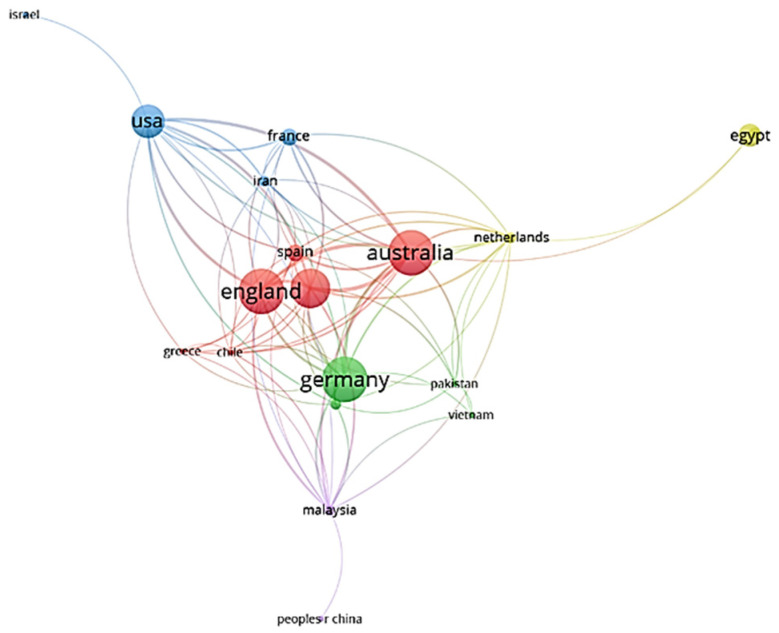
Network connection between citations and countries.

Countries in the top ranks show that economically and technologically strong countries are more active in this field. Some countries’ publications appear only as single-country publications. This shows that these countries do not collaborate with other countries. Because more than half of the publications are co-authored, multiple countries may enroll for an article. The authors’ collaboration networks show that growth is occurring in a multi-institutional and international way, utilizing the infrastructures accessible in the authors’ organizations and countries (Figure 5). After analyzing the articles under the headings of biomimicry and energy efficiency, the results were obtained in the form of three clusters: the red cluster represents Australia, Chile, Greece, and Italy; the green cluster represents the United Kingdom, France, Spain, and Turkey; and the blue cluster represents Germany, the Netherlands, and the United States. Figure 6 shows the relation between countries and citations. The five countries with the most citations are Germany (304), the United Kingdom (205), the United States (132), Italy (120), and Australia (81), respectively.

### 3.4. Analysis of Authorship

**Figure 7:**
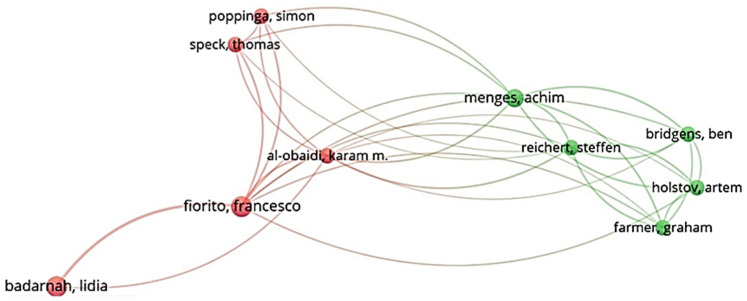
Most cited authors.

Figure 7 shows the top 10 authors with the most citations of their articles related to biomimicry and energy efficiency in buildings. According to the results obtained from the cluster network, the greatest number of citations (199), with a total link strength of 21, belongs to “Achim, Menges”. In this part of the analysis, the identification of the most influential authors was considered. The top 10 authors with the most citations and their link strength are shown in Table 2, and Table 3 shows the 10 most cited articles on biomimicry and energy efficiency. Ideas and processes related to biomimicry and energy efficiency in buildings are presented in these articles. The main focus of the articles is on the energy efficiency of building envelopes according to design approaches.

### 3.5. Analysis of Organizations

**Figure 8:**
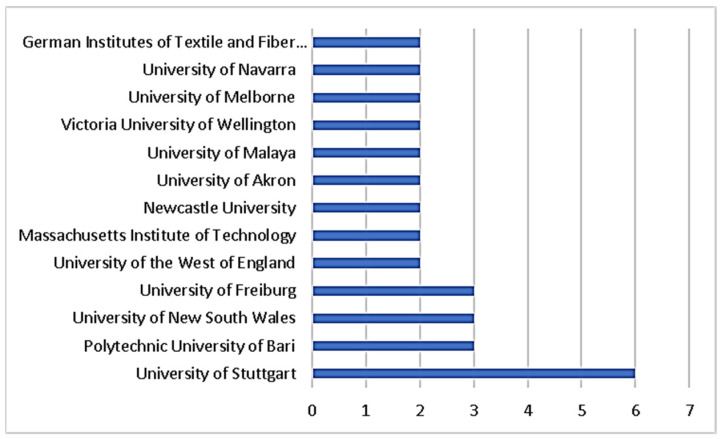
Organizational publications in the field of biomimicry and energy efficiency.

**Figure 9:**
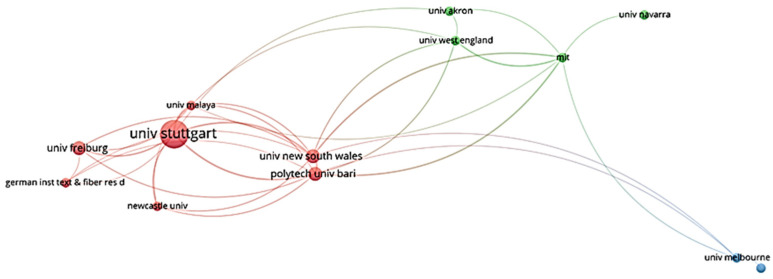
Visualization of organizations’ citation network.

The performance of organizations with two or more publications in terms of biomimicry and energy efficiency is represented by the graph bar in Figure 8. The University of Stuttgart is the most productive research center with the most publications (6) in the related research area. The Polytechnic University of Bari in Italy, the University of South Wales in the United Kingdom, and the University of Freiburg in Germany each have three publications, while the other organizations have one or two. The list of organizations that have researched in the fields of biomimicry and energy efficiency includes a diverse range of countries. According to Figure 9, Stuttgart University, with 299 citations, has the highest number; the University of Freiburg (148) and Newcastle University (88) follow.

### 3.6. Citation by Sources

**Figure 10:**
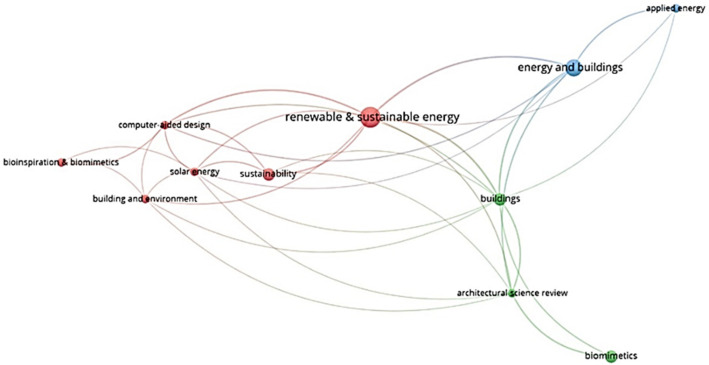
Visualization of publication sources’ citations.

The relationship between publications and citations is presented in the form of a network visualization in Figure 10. The size of the node is proportional to the number of publications, so the more articles, the larger the size of the node. The highest number of publications (5) and citations (176) belongs to the *Renewable and Sustainable Energy Reviews* journal, with total link strength of 24. It is followed by *Energy and Buildings*, *Buildings*, and *Sustainability*. The distribution of articles classified as biomimicry and energy efficiency in the field of architecture are mostly published in the journals listed in Table 4.

### 3.7. Co-Occurence of Keywords

**Figure 11:**
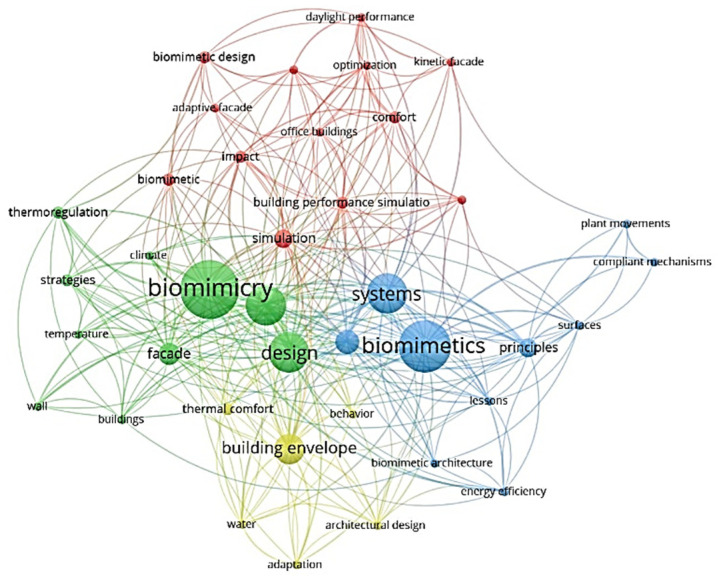
Co-occurrence of keywords.

The network diagram shows the keywords in the appropriate publications and their relationships to each other (Figure 11). The keywords analyzed were words that were repeated at least three times in the selected articles. As a result, 39 keywords were obtained in 4 groups. Relating keywords to the main content of the publications is key to revealing trends in research topics. This study found that the authors preferentially used the keywords “Biomimicry” and “Biomimetics”. It was also found that the focus of the studies is on different design strategies for thermal performance through building envelopes.

### 3.8. Content Analysis of the Selected Articles

In nature-based research, adaptation mechanisms are explored to achieve quantitative and qualitative ideas, such as the transfer of information between scales, programs, processes, or partnerships, and, as a result, a design solution is established. Solving difficulties, such as form, materials, shape, processes, and systems, in this kind of study is confined to the biological knowledge of the designer. Support for such a study by a multidisciplinary team will significantly lessen the challenge of interdisciplinary information transmission.

A content analysis was performed in this part of the research for the articles to better comprehend and remark on biologically inspired studies that altered the energy performance of buildings in the architectural literature as a consequence of multidisciplinary investigations (Table 5).

When the 47 directly related articles were examined, innovative designs for the built environment, such as energy efficiency, reductions in energy consumption, water harvesting, thermoregulation, and sensitive and responsive façades and materials, were found to be produced without the help of motors, computerized devices, or external energy sources. The common feature of all these studies is that they aim to minimize the damage to nature, reduce energy consumption, and produce energy-efficient, zero-energy buildings. In this context, it is seen that biomimetic design offers many opportunities for energy-efficient buildings. The main reason why the examined studies mostly focus on the building envelope is that this has properties that require the control of various environmental factors such as ventilation, humidity, heat, light, and mechanical stress, just like shells, the skin, and surfaces in nature. Nature has the potential to provide unlimited examples for the production of sustainable, adaptive, adaptable, and energy-efficient buildings.

**Figure 12:**
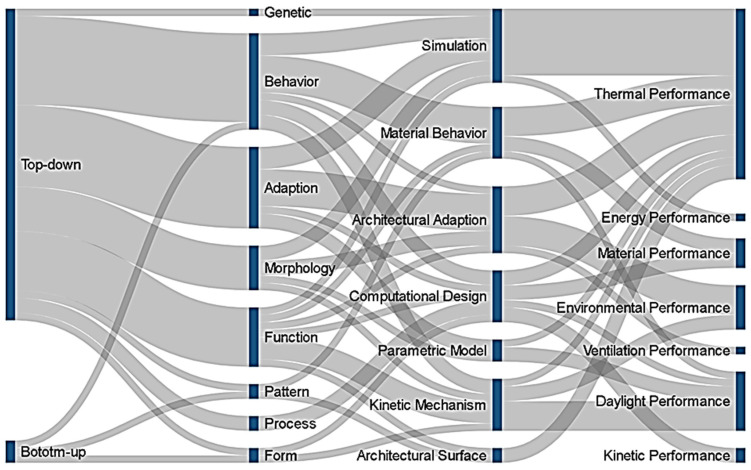
Sankey diagram of the biomimetic approach, biological phenomena, transition to architecture, and issues to be solved.

According to the Sankey diagram (Figure 12), more than 70% of the approaches to solving the energy problem in buildings are problem oriented. Concepts such as simulations, parametric models, kinetic mechanisms, and computational design are used to transfer the solutions found in nature to architectural designs. The data obtained by analyzing the biomimicry approach, the biological phenomena of nature, and the architectural research based on these phenomena and what topics they cover in energy efficiency are presented through data visualization. In this paper, “information analysis techniques” were used. It can be observed that the architectural studies interact with nature in the framework of a technological perspective and that architecture, biology, and technology are closely linked.

## 4. Conclusions

Biomimicry is a novel approach, and there has been very little research carried out in this area. The increased number of recently published articles demonstrates that there has been notable research in this area. Challenges in energy and climate change have led researchers to establish nature-based designs that demonstrate the importance of biomimicry in today’s world. The analysis of the WoS database revealed that, in recent years, there has been notable growth in the number of publications on this topic. Germany, the United Kingdom, and Australia have the most significant influence on biomimicry research. The University of Stuttgart has the greatest number of publications and citations. According to the detailed analysis of the papers, it was found that most of the studies focus on building envelopes to control various environmental factors through a problem-oriented approach. This review of articles on nature-inspired architectural studies has shown that architecture, biology, and technology are deeply entwined, and multidisciplinary studies are now inevitable.

According to these limited findings, it was found that further study in biomimicry is needed. However, in addition to the increasing trend of studies conducted in recent years, it was found that subject-oriented studies have also expanded. Based on this analysis of the studies, the systematic use of biomimicry can provide researchers with new solutions for energy-efficient architectural designs.

## Figures and Tables

**Table 1:** Publication by research area.

Research Area	Publications	Research Area	Publications
Engineering	23	Urban Studies	2
Construction and Building Technology	16	Computer Science	2
Energy and Fuels	16	Robotics	2
Science and Technology	12	Thermodynamics	2
Material Science	9	Physics	1
Architecture	6	Chemistry	1
Environmental Science and Technology	6	Instruments and Instrumentation	1

**Table 2:** Top 10 authors with the highest number of citations.

Rank	Author	Document	Citation	Total Link Strength
1	Fiorito, Francesco	4	67	27
2	Badarnah, Lidia	4	66	9
3	Menges, Achim	3	199	21
4	Reichert, Steffen	2	134	19
5	Bridgens, Ben	2	88	13
6	Holstov, Artem	2	88	13
7	Farmer, Graham	2	88	13
8	Speck, Thomas	2	118	10
9	Poppinga, Simon	2	118	10
10	Al-Obaidi, Karam M	2	76	19

**Table 3:** Top 10 highly cited papers.

Rank	Title	Year	Citation
1	“Meteorosensitive architecture: Biomimetic building skins based on materially embedded and hygroscopically enabled responsiveness” [12]	2015	90
2	“Toward a new generation of smart biomimetic actuators for architecture” [13]	2018	65
3	“Hygromorphic materials for sustainable responsive architecture” [14]	2015	59
4	“A methodology for transferring principles of plant movements to elastic systems in architecture” [15]	2015	53
5	“Toward mitigating urban heat island effects: Investigating the thermal-energy impact of bio-inspired retro-reflective building envelopes in dense urban settings” [16]	2015	53
6	“Shape morphing solar shadings: A review”	2016	49
7	“How plants inspire facades. From plants to architecture: Biomimetic principles for the development of adaptive architectural envelopes” [17]	2017	49
8	“Material capacity: Embedded responsiveness” [18]	2012	44
9	“A methodology for the generation of biomimetic design concepts” [19]	2015	43
10	“Design optimisation of solar shading systems for tropical office buildings: Challenges and future trends” [20]	2018	40

**Table 4:** List of journals with more than one publication in biomimicry and energy efficiency.

Rank	Journal	Documents	Citation	Total Link Strength
1	*Renewable and Sustainable Energy Reviews*	5	174	24
2	*Energy and Buildings*	4	66	18
3	*Buildings*	3	37	20
4	*Sustainability*	3	30	9
5	*Computer-Aided Design*	2	143	15
6	*Architectural Science Review*	2	52	14
7	*Solar Energy*	2	40	11
8	*Building and Environment*	2	26	7
9	*Applied Energy*	2	6	5
10	*Bioinspiration & Biomimetics*	2	17	4
11	*Biomimetics*	3	0	5

**Table 5:** Content analysis of articles.

Year	Description	Reference
2012	“Biomimetic responsive material systems that do not require an external energy source or any mechanical or electronic control”	[18]
2013	“A novel type of kinetic envelope design inspired by plant movements”	[21]
2014	“Design of an adaptive responsive facade based on tracking the position of the sun inspired by shrimps’ compound eyes”	[22]
2015	“An approach for generating biomimetic design ideas and water-harvesting surface designs”	[19]
2015	“Investigation of the effect of thermal energy on bio-inspired reflective building envelopes in dense urban areas”	[16]
2015	“Building systems that adapt to their environment through the usage of hygromorphic materials”	[14]
2015	“Building-shell design using smart materials that act similarly to human skin”	[23]
2015	“Responsive biomimetic building envelope with hygrometric material properties”	[12]
2015	“A novel type of kinetic envelope design inspired by plant movements for shading”	[15]
2016	“Proposing a biomimetic building envelope to reduce energy consumption, conserve materials, and increase building sustainability”	[24]
2016	“Design ideas for shape-morphing sunshades are examined, with a focus on energy-efficient smart materials and biomimetic principles”	[25]
2016	“Biomimetic design for enhancing thermal energy performance in office buildings through the use of the biomimicry approach to building energy efficiency”	[26]
2017	“Biomimetic building envelopes based on the adaptive approach”	[27]
2017	“Energy-efficient and environmentally responsive building envelope design”	[28]
2017	“Using biomimetic principles to develop energy-efficient buildings to reduce energy consumption ”	[29]
2017	“Building envelope design to reduce energy consumption”	[30]
2017	“Building systems that adapt to their surroundings through the usage of hygromorphic materials”	[31]
2017	“Developing adaptive energy-efficient building envelope inspired by plants”	[17]
2017	“Prototype of a biomimetic passive cooling panel system”	[32]
2018	“Design of an energy-efficient building envelope for office buildings based on a solar shading system”	[20]
2018	“Design of an energy-efficient building envelope based on material design without hinges for smart and adjustable exterior shading systems”	[33]
2018	“Developing parameters for reducing energy consumption through biomimetic building envelopes”	[34]
2018	“Efficient and sensitive material design for shading elements that work without stimulus inspired by plant movements”	[13]
2018	“Design of a foldable shading system without hinges”	[35]
2018	“Design of an energy-efficient office building façade”	[36]
2018	“Design of an adaptive and energy-efficient building façade”	[37]
2019	“Biomimetic approaches to zero-energy building design”	[38]
2019	“Improving thermal performance through responsive and kinetic façade design”	[39]
2019	“Environmentally sensitive building envelope design”	[40]
2019	“Environmentally sensitive biomimetic adaptive building envelope design”	[41]
2019	“Adaptive biomimetic façade design for tall glazed structures to improve energy efficiency”	[42]
2020	“Design of a biomimetic energy-efficient building”	[43]
2020	“Biomimetic design tools for building energy efficiency by managing heat through building envelopes have been developed”.	[44]
2020	“Design of a biomimetic adaptive building envelope”	[45]
2020	“Design and performance evaluation of thermo-sensitive shading prototypes”	[46]
2021	“Design of a biomimetic building envelope to improve thermal performance”	[47]
2021	“Design of biomimetic adaptable electrochromic windows to increase building energy efficiency”	[48]
2021	“Design of concrete tiles inspired by natural geometries to increase thermal performance in the building envelope”	[49]
2021	“A water-harvesting technique derived from plants”	[50]
2021	“Design of a zero-energy, nature-inspired building with high thermal comfort”	[51]
2021	“Simulation of biomimetic adaptive building envelopes that are adjusted to changing environmental circumstances”	[52]
2021	“A kinetic façade inspired by origami to increase daylight performance and energy efficiency”	[53]
2021	“Natural morphological adaptations for evaporative cooling in façade design”	[54]
2021	“Façade systems and solar panels designed on the base of automated thermal expansion, with low energy consumption and low environmental impact, without external energy sources or computerized control systems”	[55]
2021	“Surface design for evaporative exchange and temperature management”	[56]
2021	“Design of a biomimetic façade to reduce energy consumption”	[57]
2021	“Design of biomimetic building envelope systems”	[58]

## Data Availability

Data sharing not applicable.

## References

[1] IEA Energy Efficiency 2021. https://www.iea.org/reports/energy-efficiency-2021..

[2] Pawlyn M. (2016). Biomimicry in Architecture.

[3] Benyus J.M. (1997). Biomimicry Innovation Inspired by Nature.

[4] Radwana G.A.N., Osamab N. (2016). Biomimicry, an approach, for energy effecient building skin design. Procedia Environ. Sci..

[5] Vincent J., Bogatyreva O., Pahl A.K., Bogatyrev N., Bowyer A. (2005). Putting Biology into TRIZ: A Database of Biological Effects. Creat. Innov. Manag..

[6] Helms M., Vattam S.S., Goel A.K. (2009). Biologically inspired design: Process and products. Des. Stud..

[7] Cobo M., López-Herrera A.G., Herrera-Viedma E., Herrera F. (2011). Science Mapping Software Tools: Review, Analysis, and Cooperative Study Among Tools. J. Am. Soc. Inf. Sci. Technol..

[8] Waltman L., Boyack K.W., Colavizza G., van Eck N.J. (2020). A principled methodology for comparing relatedness measures for clustering publications. Quant. Sci. Stud..

[9] Coulter N., Monarch I., Konda S. (1998). Software Engineering as Seen through Its Research Literature: A Study in Co-Word Analysis. JASIS.

[10] Analytics C. (2021). Web of Science. https://clarivate.com/webofsciencegroup/solutions/web-of-science/.

[11] SankeyMATIC: A Sankey Diagram Builder for Everyone. http://sankeymatic.com/.

[12] Reichert S., Menges A., Correa D. (2015). Meteorosensitive architecture: Biomimetic building skins based on materially embedded and hygroscopically enabled responsiveness. Comput.-Aided Des..

[13] Poppinga S., Zollfrank C., Prucker O., Ruhe J., Menges A., Cheng T., Speck T. (2018). Toward a New Generation of Smart Biomimetic Actuators for Architecture. Adv. Mater..

[14] Holstov A., Bridgens B., Farmer G. (2015). Hygromorphic Materials for Sustainable Responsive Architecture. Constr. Build. Mater..

[15] Schleicher S., Lienhard J., Poppinga S., Speck T., Knippers J. (2015). A methodology for transferring principles of plant movements to elastic systems in architecture. Comput.-Aided Des..

[16] Han Y.L., Taylor J.E., Pisello A.L. (2015). Toward mitigating urban heat island effects: Investigating the thermal-energy impact of bio-inspired retro-reflective building envelopes in dense urban settings. Energy Build..

[17] Lopez M., Rubio R., Martin S., Croxford B. (2017). How plants inspire façades. From plants to architecture: Biomimetic principles for the development of adaptive architectural envelopes. Renew. Sustain. Energy Rev..

[18] Menges A., Reichert S. (2012). Material Capacity: Embedded Responsiveness. Archit. Des..

[19] Badarnah L., Kadri U. (2015). A methodology for the generation of biomimetic design concepts. Archit. Sci. Rev..

[20] Al-Masrani S., Al-Obaidi K.M., Zalin N.A., Isma M.I.A. (2018). Design optimisation of solar shading systems for tropical office buildings: Challenges and future trends. Sol. Energy.

[21] Knippers J., Jungjohann H., Scheible F., Oppe M. (2013). Bio-inspired kinetic facade for the thematic pavilion “One Ocean” EXPO 2012 in Yeosu, Korea. Bautechnik.

[22] Park J.J., Dave B. (2014). Bio-inspired Parametric Design for Adaptive Stadium Façades. Australas. J. Constr. Econ. Build. Conf. Ser..

[23] Nessim M.A. (2015). Biomimetic Architecture as a New Aproach for Energy Efficient Buildings through Smart Building Materials. J. Green Build..

[24] ElDin N.N., Abdou A., ElGawad I.A. (2016). Biomimetic Potentials for Building Envelope Adaptation in Egypt. Procedia Environ. Sci..

[25] Fiorito F., Sauchelli M., Arroyo D., Pesenti M., Imperadori M., Masera G., Ranzi G. (2016). Shape morphing solar shadings: A review. Renew. Sustain. Energy Rev..

[26] Zuazua-Ros A., Martin-Gomez C., Bermejo-Busto J., Vidaurre-Arbizu M., Baquero E., Miranda R. (2016). Thermal energy performance in working-spaces from biomorphic models: The tuna case in an office building. Build. Simul..

[27] Al-Obaidi K.M., Ismail M.A., Hussein H., Rahman A.M.A. (2017). Biomimetic building skins: An adaptive approach. Renew. Sustain. Energy Rev..

[28] Badarnah L. (2017). Form follows environment: Biomimetic approaches to building envelope design for environmental adaptation. Buildings.

[29] Chayaamor-Heil N., Hannachi-Belkadi N. (2017). Towards a Platform of Investigative Tools for Biomimicry as a New Approach for Energy-Efficient Building Design. Buildings.

[30] Fecheyr-Lippens D., Bhiwapurkar P. (2017). Applying biomimicry to design building envelopes that lower energy consumption in a hot-humid climate. Archit. Sci. Rev..

[31] Holstov A., Farmer G., Bridgens B. (2017). Sustainable materialisation of responsive architecture. Sustainability.

[32] Zuazua-Ros A., Gomez C.M., Ramos J.C., Bermejo-Busto J. (2017). Towards cooling systems integration in buildings: Experimental analysis of a heat dissipation panel. Renew. Sustain. Energy Rev..

[33] Korner A., Born L., Mader A., Sachse R., Saffarian S., Westermeier A.S., Poppinga S., Bischoff M., Gresser G.T., Milwich M. (2018). Flectofold-a biomimetic compliant shading device for complex free form facades. Smart Mater. Struct..

[34] Mohamed A.S.Y. (2018). Biomimetic Architecture: Creating a Passive Defense System in Building Skin to Solve Zero Carbon Construction Dilemma. Eqa-Int. J. Environ. Qual..

[35] Schieber G., Born L., Bergmann P., Korner A., Mader A., Saffarian S., Betz O., Milwich M., Gresser G.T., Knippers J. (2018). Hindwings of insects as concept generator for hingeless foldable shading systems. Bioinspir. Biomim..

[36] Webb M., Aye L., Green R. (2018). Simulation of a biomimetic façade using TRNSYS. Appl. Energy.

[37] Xing Y.G., Jones P., Bosch M., Donnison I., Spear M., Ormondroyd G. (2018). Exploring design principles of biological and living building envelopes: What can we learn from plant cell walls?. Intell. Build. Int..

[38] Cuce E., Nachan Z., Cuce P.M., Sher F., Neighbour G.B. (2019). Strategies for ideal indoor environments towards low/zero carbon buildings through a biomimetic approach. Int. J. Ambient. Energy.

[39] Hosseini S.M., Mohammadi M., Rosemann A., Schroder T., Lichtenberg J. (2019). A morphological approach for kinetic facade design process to improve visual and thermal comfort: Review. Build. Environ..

[40] Khosromanesh R., Asefi M. (2019). Form-finding mechanism derived from plant movement in response to environmental conditions for building envelopes. Sustain. Cities Soc..

[41] Kuru A., Oldfield P., Bonser S., Fiorito F. (2019). Biomimetic adaptive building skins: Energy and environmental regulation in buildings. Energy Build..

[42] Sheikh W.T., Asghar Q. (2019). Adaptive biomimetic facades: Enhancing energy efficiency of highly glazed buildings. Front. Archit. Res..

[43] Imani N., Vale B. (2020). A framework for finding inspiration in nature: Biomimetic energy efficient building design. Energy Build..

[44] Imani N., Vale B. (2020). The Development of a Biomimetic Design Tool for Building Energy Efficiency. Biomimetics.

[45] Kuru A., Oldfield P., Bonser S., Fiorito F. (2020). A Framework to Achieve Multifunctionality in Biomimetic Adaptive Building Skins. Buildings.

[46] Yoon J., Bae S. (2020). Performance Evaluation and Design of Thermo-Responsive SMP Shading Prototypes. Sustainability.

[47] Abdel-Rahman W.S.M. (2021). Thermal performance optimization of parametric building envelope based on bio-mimetic inspiration. Ain Shams Eng. J..

[48] Bui D.K., Nguyen T.N., Ghazlan A., Ngo T.D. (2021). Biomimetic adaptive electrochromic windows for enhancing building energy efficiency. Appl. Energy.

[49] Hershcovich C., van Hout R., Rinsky V., Laufer M., Grobman Y.J. (2021). Thermal performance of sculptured tiles for building envelopes. Build. Environ..

[50] Jalali S., Aliabadi M., Mahdavinejad M. (2021). Learning from plants: A new framework to approach water-harvesting design concepts. Int. J. Build. Pathol. Adapt..

[51] Jankovic L., Carta S. (2021). BioZero-Designing Nature-Inspired Net-Zero Building. Sustainability.

[52] Kuru A., Oldfield P., Bonser S., Fiorito F. (2021). Performance prediction of biomimetic adaptive building skins: Integrating multifunctionality through a novel simulation framework. Sol. Energy.

[53] Luan L.T., Thang L.D., Hung N.M., Nguyen Q.H., Nguyen-Xuan H. (2021). Optimal design of an Origami-inspired kinetic facade by balancing composite motion optimization for improving daylight performance and energy efficiency. Energy.

[54] Peeks M., Badarnah L. (2021). Textured Building Facades: Utilizing Morphological Adaptations Found in Nature for Evaporative Cooling. Biomimetics.

[55] Petriccione L., Fulchir F., Chinellato F. (2021). Applied innovation: Technological experiments on biomimetic facade systems and solar panels. Techne-J. Technol. Archit. Environ..

[56] Rupp A., Gruber P. (2021). Bio-inspired evaporation from shaped interfaces: An experimental study. Bioinspir. Biomim..

[57] Webb M. (2021). Biomimetic building facades demonstrate potential to reduce energy consumption for different building typologies in different climate zones. Clean Technol. Environ. Policy.

[58] Cruz E., Hubert T., Chancoco G., Naim O., Chayaamor-Heil N., Cornette R., Menezo C., Badarnah L., Raskin K., Aujard F. (2021). Design processes and multi-regulation of biomimetic building skins: A comparative analysis. Energy Build..

